# Activation of K_Ca_3.1 K^+^ Channel Suppresses COX2- and ARG1-Dependent Immunosuppressive Metabolic Programs in THP-1-Derived M_2_-like Macrophages

**DOI:** 10.3390/ijms27198477

**Published:** 2026-09-23

**Authors:** Junko Kajikuri, Ayano Kitahara, Miki Matsui, Yasuhiro Maeda, Hiroaki Kito, Yohei Yamaguchi, Ryo Otsuka, Susumu Ohya

**Affiliations:** 1 Department of Pharmacology, Graduate School of Medical Sciences, Nagoya City University, Nagoya 467-8601, Japan; kajikuri@med.nagoya-cu.ac.jp (J.K.); kita.aya@med.nagoya-cu.ac.jp (A.K.); c241739@ed.nagoya-cu.ac.jp (M.M.); kito@med.nagoya-cu.ac.jp (H.K.); y_yamagu@med.nagoya-cu.ac.jp (Y.Y.); c241905@ed.nagoya-cu.ac.jp (R.O.); 2 Omics Analysis Laboratory, Open Facility Center, Fujita Health University, Toyoake 470-1192, Japan; ymaeda@fujita-hu.ac.jp

**Keywords:** M_2_-like macrophage, K_Ca_3.1, K^+^ channel, tumor microenvironment, COX2, ARG1, PGE_2_, arginine, elevated extracellular K^+^ concentration

## Abstract

M_2_-like macrophages contribute to the establishment of immunosuppressive microenvironments through coordinated activation of inflammatory and metabolic programs. Cyclooxygenase-2 (COX2) catalyzes prostaglandin E_2_ (PGE_2_) production, whereas arginase-1 (ARG1) hydrolyzes L-arginine (ARG) to ornithine (ORN) and urea. However, the ion channel-dependent mechanisms governing these immunometabolic pathways remain poorly defined. Here, we examined the role of the Ca^2+^-activated K^+^ channel K_Ca_3.1 in regulating COX2 and ARG1 in THP-1-derived M_2_-like macrophages. COX2 and ARG1 were markedly upregulated during M_2_-like differentiation, whereas pharmacological activation of K_Ca_3.1 with SKA-121 significantly reduced their mRNA and protein expression. K_Ca_3.1 activation also decreased PGE_2_ production, increased extracellular ARG, and reduced ORN accumulation. Exposure to elevated extracellular K^+^ concentration ([K^+^]_e_) further enhanced COX2 and ARG1 expression, increased PGE_2_ production, and shifted ARG metabolism toward ORN formation; each of these responses was attenuated by K_Ca_3.1 activation. Pharmacological inhibition of ERK or CREB signaling suppressed both COX2 and ARG1 expression, whereas inhibition of JNK, AP-1, NOX2, or NRF2 had limited effects. CREB2 knockdown similarly reduced COX2 and ARG1 under both basal and elevated [K^+^]_e_ conditions. These findings demonstrate that K_Ca_3.1 activation negatively regulates COX2- and ARG1-associated immunometabolic programs in THP-1-derived M_2_-like macrophages and suggest a potential role for K_Ca_3.1 in modulating macrophage-associated inflammatory and metabolic responses.

## 1. Introduction

The tumor microenvironment (TME) is a heterogeneous milieu composed of malignant, stromal, and infiltrating immune cells that engage in complex reciprocal interactions [1]. Tumor-associated macrophages (TAMs), many of which exhibit M_2_-like features, are among the most abundant immune populations in solid tumors. Their accumulation is frequently associated with poor clinical outcomes, and they promote tumor progression through immune suppression, angiogenesis, tissue remodeling, and support of cancer cell survival [2,3]. These protumorigenic activities are mediated not only by cytokines and chemokines but also by lipid mediators and amino acid-metabolic pathways [3,4]. The TME is additionally characterized by metabolic stressors, including lactate accumulation, extracellular acidosis, hypoxia, and nutrient deprivation, which collectively promote tumor growth, immune evasion, and therapeutic resistance [5,6]. Elevated extracellular K^+^ concentration ([K^+^]_e_) has more recently emerged as another important feature of the TME [7]. Necrotic and dying tumor cells release intracellular K^+^ into the extracellular space, thereby perturbing ionic homeostasis and altering antitumor immune cell function.

The intermediate-conductance Ca^2+^-activated K^+^ channel K_Ca_3.1, encoded by KCNN4, is an important regulator of immune cell activation, cytokine/chemokine production, and cellular metabolism [8,9]. We previously demonstrated that pharmacological activation of K_Ca_3.1 downregulated interleukin-8 (IL-8), IL-10, and C-C motif chemokine ligand 2 (CCL2) in THP-1-derived M_2_-like macrophages (M_2_-MACs) [10,11]. These mediators contribute to the recruitment and functional regulation of immunosuppressive cell populations in pathological tissues [12,13], supporting a broader role for K_Ca_3.1 in controlling macrophage-derived inflammatory and immunoregulatory signals. In those studies, elevated [K^+^]_e_ significantly increased IL-8, IL-10, and CCL2 expression and production in M_2_-MACs, whereas K_Ca_3.1 activation attenuated their induction [10,11]. These observations suggest that K_Ca_3.1 counteracts elevated [K^+^]_e_-induced inflammatory responses. Whether K_Ca_3.1 also regulates immunometabolic enzymes in M_2_-MACs, however, remains unknown. This question is important because M_2_-MACs contribute to immune suppression through metabolic mediators as well as cytokines and chemokines [3,4]. Cyclooxygenase-2 (COX2), also known as prostaglandin-endoperoxide synthase 2 (PTGS2), catalyzes the rate-limiting step in prostaglandin biosynthesis and promotes the production of prostaglandin E_2_ (PGE_2_), a lipid mediator with potent immunoregulatory and tumor-promoting activities [14]. In parallel, arginase-1 (ARG1) hydrolyzes L-arginine (ARG) to ornithine (ORN) and urea [15]. ARG1-dependent depletion of ARG impairs T cell proliferation and effector functions, whereas ORN provides a precursor for polyamine and proline biosynthesis, thereby supporting tissue remodeling and tumor progression [14,15]. Thus, the COX2–PGE_2_ and ARG1–ARG/ORN pathways represent complementary immunometabolic programs through which M_2_-MACs may shape pathological microenvironments.

In the present study, we investigated whether K_Ca_3.1 activation regulates COX2 and ARG1 expression in M_2_-MACs and whether such regulation is accompanied by changes in PGE_2_ production and ARG metabolism. We further examined the effects of elevated [K^+^]_e_ and explored the signaling pathways responsible for COX2 and ARG1 induction. Our results show that K_Ca_3.1 activation with SKA-121 suppresses both basal and elevated [K^+^]_e_-induced COX2 and ARG1 expression and attenuates the associated changes in PGE_2_, ARG, and ORN. These findings extend the known role of K_Ca_3.1 from cytokine and chemokine regulation to the control of macrophage immunometabolism.

## 2. Results

### 2.1. K_Ca_3.1 Activation Suppresses COX2 Expression and PGE_2_ Production in M_2_-MACs

We previously reported that CD163 and ARG1, commonly used markers of human M_2_-MACs, were upregulated in THP-1-differentiated M_2_-MACs and that K_Ca_3.1 activation elicited membrane hyperpolarization in these cells [10]. In the present study, quantitative real-time PCR and western blotting showed that COX2 expression was markedly higher in M_2_-MACs than in undifferentiated THP-1 cells (*n* = 4 for each, *p* < 0.01) (Figure 1A, Supplementary Figure S1A,B), indicating robust induction of COX2 during M_2_-like differentiation. Treatment with the K_Ca_3.1 activator SKA-121 (10 μM) for 24 h significantly reduced COX2 mRNA expression (*n* = 4, *p* < 0.01) (Figure 1B). Consistent with this finding, SKA-121 treatment for 48 h decreased COX2 protein abundance (*n* = 4, *p* < 0.01) (Figure 1C,D) and reduced the PGE_2_ concentration in the culture medium (*n* = 4, *p* < 0.01) (Figure 1E). A comparable reduction in PGE_2_ was observed with the selective COX2 inhibitor celecoxib (1 μM) (Figure 1F). Together, these results indicate that K_Ca_3.1 activation suppresses the COX2–PGE_2_ axis in M_2_-MACs.

To determine whether the suppressive effects of SKA-121 on COX2 expression were mediated through K_Ca_3.1, siRNA-mediated inhibition of K_Ca_3.1 was performed in M_2_-MACs. Transfection with K_Ca_3.1-specific siRNA (siK_Ca_3.1) significantly reduced K_Ca_3.1 mRNA expression compared with control siRNA (siCont) (Supplementary Figure S2A). In cells transfected with control siRNA, SKA-121 significantly decreased COX2 expression (Supplementary Figure S2B). In contrast, following K_Ca_3.1 knockdown, SKA-121 failed to reduce it (Supplementary Figure S2D). These results indicate that the inhibitory effects of SKA-121 on COX2 expression support a K_Ca_3.1-mediated mechanism of action.

### 2.2. K_Ca_3.1 Activation Attenuates Elevated Extracellular K^+^ ([K^+^]_e_)-Induced COX2 Expression in M_2_-MACs

Elevated [K^+^]_e_ is increasingly recognized as an ionic feature of the TME [7]. In vivo K^+^ imaging has shown that [K^+^]_e_ can reach an average concentration of approximately 30 mM within tumors [16]. We previously found that exposure of M_2_-MACs to 35 mM [K^+^]_e_ increased intracellular K^+^ by approximately 1.3-fold [11]. We therefore examined the effect of elevated [K^+^]_e_ on COX2 expression. Addition of 30 mM KCl, which increased the final [K^+^]_e_ to approximately 35 mM, increased COX2 mRNA expression by approximately five-fold after 24 h (*n* = 4, *p* < 0.01) (Figure 2A). No significant changes in COX2 mRNA expression were found by addition of 10 and 20 mM KCl after 24 h (*n* = 4, *p* > 0.05) (Supplementary Figure S1E). Co-treatment with SKA-121 (10 μM) significantly attenuated this increase (*n* = 4, *p* < 0.01). Similarly, elevated [K^+^]_e_ increased COX2 protein abundance and PGE_2_ production after 48 h, and both responses were suppressed by SKA-121 (Figure 2B–D). These findings indicate that elevated [K^+^]_e_ activates the COX2–PGE_2_ pathway in M_2_-MACs and that K_Ca_3.1 activation counteracts this response.

### 2.3. K_Ca_3.1 Activation Suppresses ARG1 Expression and Alters ARG Metabolism in M_2_-MACs

ARG1 expression was also markedly higher in M_2_-MACs than in undifferentiated THP-1 cells (*n* = 4, *p* < 0.01) (Figure 3A, Supplementary Figure S1C,D), confirming induction of ARG1 during M_2_-like differentiation. Treatment with SKA-121 (10 μM) for 24 h significantly reduced ARG1 mRNA expression (*n* = 4, *p* < 0.01) (Figure 3B), and treatment for 48 h decreased ARG1 protein abundance (*n* = 4, *p* < 0.01) (Figure 3C,D). In cells transfected with control siRNA, SKA-121 significantly decreased ARG1 mRNA expression (Supplementary Figure S2C). In contrast, following K_Ca_3.1 knockdown, SKA-121 failed to reduce ARG1 expression (Supplementary Figure S2E). These results indicate that the inhibitory effects of SKA-121 on ARG1 expression support a K_Ca_3.1-mediated mechanism of action.

These changes were accompanied by an increase in extracellular ARG and a decrease in extracellular ORN (*n* = 4, *p* < 0.01) (Figure 3E,F). The ARG1 inhibitor numidargistat (10 μM) produced similar metabolic changes (Supplementary Figure S3A,B), supporting the interpretation that SKA-121 reduces ARG1-dependent ARG catabolism. Because ARG is also a substrate for nitric oxide synthases (NOSs), which convert ARG to L-citrulline (CIT) [17], we measured CIT in the culture medium. SKA-121 did not significantly alter CIT concentrations (*n* = 4, *p* > 0.05) (Supplementary Figure S3C). Consistent with this result, NOS2 mRNA expression was very low (less than 0.01 relative to ACTB). We further examined whether the reduction in ARG1 expression induced by SKA-121 was accompanied by a functional decrease in arginase enzymatic activity. Arginase activity was measured in cell lysates from M_2_-MACs using a colorimetric assay based on urea production from L-arginine. Treatment with SKA-121 significantly reduced arginase activity (*n* = 4, *p* < 0.01) (Figure 3G). Thus, under the present experimental conditions, K_Ca_3.1 activation selectively altered the ARG1-associated branch of ARG metabolism without measurably affecting the NOS-associated branch.

### 2.4. K_Ca_3.1 Activation Attenuates Elevated [K^+^]_e_-Induced ARG1 Expression in M_2_-MACs

Exposure to elevated [K^+^]_e_ for 24 h increased ARG1 mRNA expression by more than five-fold, and this induction was significantly attenuated by SKA-121 treatment (10 μM; *n* = 4, *p* < 0.01) (Figure 4A). No significant changes in ARG1 mRNA expression were found by addition of 10 and 20 mM KCl (*n* = 4, *p* > 0.05) (Supplementary Figure S1F). Elevated [K^+^]_e_ also increased ARG1 protein abundance after 48 h, whereas co-treatment with SKA-121 suppressed this response (*n* = 4, *p* < 0.01) (Figure 4B,C). Consistent with reduced ARG1 activity, SKA-121 restored ARG concentrations and decreased ORN accumulation under elevated [K^+^]_e_ conditions (*n* = 4, *p* < 0.01) (Figure 4D,E). Neither elevated [K^+^]_e_ alone nor co-treatment with SKA-121 significantly altered CIT concentrations (*n* = 4, *p* > 0.05) (Supplementary Figure S3D). To determine whether the upregulation of ARG1 under elevated [K^+^]_e_ conditions was associated with increased enzymatic activity, arginase activity was measured in M_2_-MACs. Exposure to elevated [K^+^]_e_ significantly increased arginase activity compared with control conditions (*n* = 4, *p* < 0.01) (Figure 4F). In contrast, treatment with SKA-121 significantly attenuated the elevated [K^+^]_e_-induced increase in arginase activity (*n* = 4, *p* < 0.01) (Figure 4F). These results indicate that elevated [K^+^]_e_ enhances ARG1-associated ARG catabolism in M_2_-MACs and that K_Ca_3.1 activation counteracts this metabolic response.

### 2.5. ERK and CREB Signaling Are Involved in COX2–PGE_2_ Axis Regulation Under Basal and Elevated [K^+^]_e_ Conditions

To identify signaling pathways involved in COX2 regulation, we examined the effects of pharmacological inhibitors and siRNA-mediated knockdown. Under basal [K^+^]_e_ conditions (5 mM), inhibition of ERK with SCH772984 (1 μM) or pan-CREB activity with 666-15 (1 μM) significantly reduced COX2 mRNA expression and PGE_2_ production (*n* = 4, *p* < 0.01) (Figure 5A,C). By contrast, inhibition of JNK with SP600125 (1 μM), AP-1 with T5224 (10 μM), NOX2 with GSK2795039 (10 μM), or NRF2 with ML385 (5 μM) had little effect on COX2 mRNA expression (*n* = 4, *p* > 0.05) (Figure 5A). Among the CREB/ATF family members examined previously, CREB2/ATF4 was the predominant transcript in M_2_-MACs [11]. Although ATF2 can bind CRE-containing elements in the COX2 promoter [18], ATF2 was expressed at relatively low levels in M_2_-MACs. Selective knockdown of CREB2 reduced COX2 mRNA expression and PGE_2_ production (*n* = 4, *p* < 0.01) (Figure 5B,D), whereas ATF2 knockdown showed limited effects (*n* = 4, *p* > 0.05) (Figure 5B). The knockdown efficiencies were approximately 50% (Supplementary Figure S4). CCAAT/enhancer-binding protein β (CEBPB) has been implicated in the transcriptional regulation of COX2 and ARG1 in myeloid-derived suppressor cells [19]; however, CEBPB knockdown did not significantly alter COX2 expression in M_2_-MACs (*n* = 4, *p* > 0.05) (Figure 5B). Moreover, SKA-121, SCH772984, and 666-15 did not significantly reduce CEBPB mRNA expression under either basal or elevated [K^+^]_e_ conditions (*n* = 4, *p* > 0.05) (Supplementary Figure S5). These findings implicate ERK and CREB2 in the regulation of the COX2–PGE_2_ pathway in M_2_-MACs.

Under elevated [K^+^]_e_ conditions, inhibition of ERK or pan-CREB activity markedly reduced COX2 mRNA expression and PGE_2_ production (*n* = 4, *p* < 0.01) (Figure 5E,G). CREB2 knockdown similarly attenuated elevated [K^+^]_e_-induced COX2 expression and PGE_2_ production (*n* = 4, *p* < 0.01) (Figure 5F,H), whereas knockdown of ATF2 or CEBPB had no significant effect (*n* = 4, *p* > 0.05) (Figure 5F). These results support the ERK–CREB2 axis as a major regulatory pathway for COX2 induction in M_2_-MACs, particularly under elevated [K^+^]_e_.

### 2.6. ERK and CREB2 Signaling Regulate ARG1 Expression and ARG Metabolism

We next examined the signaling mechanisms regulating ARG1 expression and ARG metabolism. Under basal [K^+^]_e_ conditions, inhibition of ERK or pan-CREB activity significantly reduced ARG1 mRNA expression (*n* = 4, *p* < 0.01) (Figure 6A). Both inhibitors increased extracellular ARG and decreased ORN (*n* = 4, *p* < 0.01) (Figure 6C,D), consistent with reduced ARG1-associated metabolic activity. CREB2 knockdown produced similar effects, reducing ARG1 expression, increasing ARG, and decreasing ORN (*n* = 4, *p* < 0.01), whereas knockdown of ATF2 or CEBPB had little effect (*n* = 4, *p* > 0.05) (Figure 6B,E,F).

Under elevated [K^+^]_e_ conditions, inhibition of ERK or CREB markedly suppressed ARG1 induction (*n* = 4, *p* < 0.01) (Figure 6G). These inhibitors also restored ARG concentrations and reduced ORN accumulation (*n* = 4, *p* < 0.01) (Figure 6I,J). CREB2 knockdown likewise attenuated elevated [K^+^]_e_-induced ARG1 expression and normalized the ARG/ORN profile (*n* = 4, *p* < 0.01) (Figure 6H,K,L).

In our previous study [10], we demonstrated that SKA-121 treatment attenuated ERK phosphorylation, whereas exposure to [K^+^]_e_ enhanced ERK phosphorylation in M_2_-MACs. However, the effects of SKA-121 and elevated [K^+^]_e_ on CREB2 expression were not fully examined. Therefore, we investigated CREB2 expression under these conditions in the present study. Treatment with SKA-121 significantly decreased CREB2 mRNA and protein expression (*n* = 4, *p* < 0.01) (Figure 7A–C). In contrast, exposure to elevated [K^+^]_e_ significantly increased CREB2 mRNA and protein expression (*n* = 4, *p* < 0.01) (Figure 7D–F). Notably, SKA-121 treatment significantly attenuated the elevated [K^+^]_e_-induced increase in CREB2 protein expression (*n* = 4, *p* < 0.01) (Figure 7D–F), suggesting that CREB2 may act downstream of ERK signaling under these conditions. Collectively, these data indicate that the ERK–CREB2 axis coordinately regulates the COX2–PGE_2_ and ARG1–ARG/ORN programs in M_2_-MACs.

Neither pharmacological inhibition of ERK or CREB nor CREB2 knockdown significantly altered CIT concentrations under basal or elevated [K^+^]_e_ conditions (Supplementary Figure S6). Because tryptophan (TRP) metabolism also contributes to immune suppression in the TME [20], we measured TRP and kynurenine (KYN). SKA-121 did not significantly alter either metabolite under basal or elevated [K^+^]_e_ conditions (Supplementary Figure S7), and the transcriptional levels of the related enzymes IDO1, IDO2, and TDO2 were very low (less than 0.01 relative to ACTB).

## 3. Discussion

The present study identifies K_Ca_3.1 activation as a negative regulator of two immunometabolic pathways in M_2_-MACs: the COX2–PGE_2_ axis and ARG1-dependent ARG catabolism. COX2 and ARG1 were markedly induced during M_2_-like differentiation, and pharmacological activation of K_Ca_3.1 with SKA-121 reduced their mRNA and protein expression (Figure 1 and Figure 3, Supplementary Figure S1). These molecular changes were accompanied by corresponding functional metabolic effects: reduced PGE_2_ production and arginase activity, increased extracellular ARG, and decreased ORN accumulation. Although polyamines were not directly measured, the reduction in ORN is consistent with decreased substrate availability for downstream polyamine and proline biosynthesis. Thus, K_Ca_3.1 activation coordinately suppresses two distinct macrophage programs with the potential to support immune suppression and tissue remodeling.

A second major finding is that elevated [K^+^]_e_ enhanced both COX2 and ARG1 expression (Figure 2 and Figure 4). The accompanying increase in PGE_2_ production and arginase activity, decrease in extracellular ARG, and accumulation of ORN indicate activation of both PG synthesis and ARG1-dependent ARG catabolism. Elevated [K^+^]_e_ occurs in necrotic tissues and tumors, where loss of membrane integrity releases intracellular [K^+^] ([K^+^]_i_) into the extracellular compartment. In the present study, elevated [K^+^]_e_ should therefore be regarded as an experimentally defined K^+^-rich condition designed to model localized regions of the TME associated with extensive tumor cell death rather than as a uniform ionic condition throught the entire TME. Our findings support the concept that elevated [K^+^]_e_ may function not only as a suppresor of cytotoxic T cell activity but also as a microenvironmental signal that reinforces immunometabolic programs in macrophages. Importantly, SKA-121 attenuated these elevated [K^+^]_e_-induced responses, suggesting that K_Ca_3.1 activation can counteract K^+^-driven macrophage immunometabolic reprogramming.

The coordinated regulation of COX2 and ARG1 is immunologically relevant because PGE_2_ production and ARG depletion suppress antitumor immunity through distinct but complementary mechanisms [21,22,23,24]. PGE_2_ inhibits cytotoxic T cell responses, promotes M_2_-MAC polarization, and enhances the suppressive activity of myeloid-derived suppressor cells through EP2 and EP4 receptors [21,22]. In contrast, ARG1 reduces ARG availability, thereby impairing T cell proliferation, activation, and effector function [23,24]. Thus, simultaneous suppression of the COX2–PGE_2_ and ARG1-dependent ARG metabolic pathways may reduce the ability of M_2_-MACs to establish an immunosuppressive metabolic environment. However, because macrophage-mediated suppression of T cell function was not directly assessed in the present study, our findings demonstrate regulation of macrophage immunometabolic pathways rather than direct restoration of antitumor immune responses.

Mechanistically, the pharmacological and gene-silencing experiments identify the ERK–CREB2 axis as an important regulator of both COX2 and ARG1 in M_2_-MACs. Inhibition of ERK or CREB, as well as CREB2 knockdown, reduced COX2 and ARG1 expression and attenuated the associated changes in PGE_2_ and ARG/ORN under both basal and elevated [K^+^]_e_ conditions (Figure 5 and Figure 6). JNK–c-JUN, NOX2, and NRF2 signaling can regulate COX2 and/or ARG1 in other cellular contexts [25,26,27], but inhibition of these pathways had limited effects in the present model, suggesting context-dependent pathway utilization. The present findings are also consistent with our previous observation that K_Ca_3.1 activation suppresses CCL2 through an ERK–CREB2-dependent mechanism in THP-1-derived M_2_-MACs [11]. K_Ca_3.1 activation alters membrane potential, Ca^2+^ signaling, and [K^+^]_i_ homeostasis [10,11], each of which may influence kinase activity and transcriptional regulation. Nevertheless, the current data do not establish whether K_Ca_3.1 activation suppresses ERK-CREB2 signaling directly or through secondary changes in ion homeostasis. Direct measurement of ERK and CREB2 activation, together with promoter occupancy studies, will be required to define this mechanism.

Glycogen synthase kinase 3 (GSK3)-dependent phosphorylation of CREB2 at Ser214 promotes its proteasomal degradation [28], suggesting the possibility that K_Ca_3.1 regulates CREB2 through a GSK3β pathway. K_Ca_3.1 has been linked to PI3K–AKT–GSK3β signaling in astrocytes [29]. However, inhibition of GSK3β with TWS119 (1 μM) did not reproduce the effects of SKA-121 on COX2 or ARG1 expression, PGE_2_ production, or the ARG/ORN profile (Supplementary Figure S8), arguing against GSK3β as the principal mediator in M_2_-MACs. We also examined pyruvate kinase M2 (PKM2), which contains a K^+^-binding site and has been implicated in ERK–COX2 signaling and interstitial K^+^ homeostasis in tumors [30,31]. PKM2 mRNA was markedly induced during M_2_-like differentiation and reduced by SKA-121 under both basal and elevated [K^+^]_e_ conditions (Supplementary Figure S9A,B). Nevertheless, PKM2 inhibition did not significantly alter COX2 expression. By contrast, PKM2 inhibition reduced ARG1 expression selectively under elevated [K^+^]_e_ conditions (Supplementary Figure S9C,D). These results suggest that PKM2 may contribute to elevated [K^+^]_e_-induced ARG1 regulation but is not required for COX2 induction. This context-dependent role is compatible with emerging evidence that, in addition to promoting inflammatory M_1_-like metabolism, PKM2 can support TAM phenotypes [30,31,32].

ARG restriction can induce compensatory expression of cationic amino acid transporters, including solute carrier 7A1 (cationic amino acid transporter 1) (SLC7A1/CAT1), SLC7A2/CAT2, SLC7A3/CAT3, and SLC7A6 [33]. Among these transporters, SLC7A2 was the predominant transcript in M_2_-MACs. The relative expression values normalized to ACTB were 0.067 ± 0.002 for SLC7A1, 0.979 ± 0.045 for SLC7A2, under 0.0005 for SLC7A3, and 0.040 ± 0.001 for SLC7A6 (*n* = 4). Neither elevated [K^+^]_e_ nor SKA-121 significantly altered their expression (Supplementary Figure S10). These findings suggest that the observed changes in extracellular ARG and ORN primarily reflect altered ARG1 activity rather than transcriptional regulation of SLC7A2. However, transporter activity was not measured directly, and the contribution of SLC7A2 to ARG flux in M_2_-MACs remains to be determined.

Several miRNAs have been implicated in the promotion of M_2_ macrophage polarization [34]. Among them, miR-223-3p is considered an important regulator of the dynamic balance between M_1_- and M_2_-MAC phenotypes. Its inhibition or downregulation has been reported to promote M_1_ polarization while suppressing M_2_ polarization [35,36]. In the present study, overexpression of miR-223-3p using a synthetic miR-223-3p mimic did not significantly affect the transcriptional levels of COX2 or ARG1 (Supplementary Figure S11A–C). Furthermore, treatment with SKA-121 for 24 h did not alter miR-223-3p expression (Supplementary Figure S11D). These findings suggest that miR-223-3p is unlikely to be directly involved in the SKA-121-mediated regulation of COX2 and ARG1 expression in M_2_-MACs.

The present findings may also be relevant to endometriosis and endometriosis-associated ovarian cancer, which are characterized by macrophage-rich inflammatory microenvironments [37,38]. The COX2–PGE_2_ axis contributes to inflammation and immune suppression, while ARG1-dependent ARG metabolism may further shape the immunometabolic environment of endometriotic lesions [39,40]. [K^+^]_e_ elevation has not yet been directly established in endometriotic lesions, and disease-specific experimental validation will therefore be required [41]. Nevertheless, the ability of K_Ca_3.1 activation to suppress COX2, PGE_2_, and ARG metabolism provides a rationale for investigating this pathway in macrophage-rich gynecological disorders.

Our previous findings that K_Ca_3.1 activation suppresses IL-8, IL-10, and CCL2 in THP-1-derived M_2_-MACs support a broader role for K_Ca_3.1 in limiting macrophage-derived protumorigenic and immunoregulatory mediators [10,11]. Taken together, the available data suggest that K_Ca_3.1 activation can coordinately modulate cytokine, chemokine, lipid-mediator, and amino acid-metabolic programs. However, disease-specific and cell type-specific validation is essential before K_Ca_3.1 activators can be considered therapeutic candidates.

Several limitations of the present study should be acknowledged. First, validation using primary human monocyte-derived macrophages, TAMs, and in vivo systems will be necessary to establish the physiological and pathological relevance of the K_Ca_3.1–COX2/ARG1 metabolic pathway. Second, the effects of SKA-121 treatment on cancer cell growth and macrophage-mediated immunosuppression were not directly assessed. Third, although changes in ORN levels were used as an indicator of ARG1-associated metabolism, downstream metabolites, including polyamines, proline, and urea, were not measured. Finally, promoter-reporter assays, chromatin immunoprecipitation, and rescue experiments will be required to determine whether CREB2 directly regulates COX2 and ARG1 transcription and to elucidate how K_Ca_3.1 activity converges on the ERK–CREB2 signaling axis.

## 4. Materials and Methods

### 4.1. Materials and Reagents

RPMI 1640 (Product code: 189-02025), DNase-free RNase (313-01461), and Penicillin-Streptomycin Solution (×100) (168-23191) were purchased from FUJIFILM Wako Pure Chemicals (Osaka, Japan). Fetal bovine serum (FBS) (Product code: 172012), 12-myristate 13-acetate (PMA) (P1585), and phosphatase inhibitor cocktails (P5726, P0044) were from Sigma-Aldrich (St. Louis, MO, USA). SP600125 (HY-12041), SKA-121 (HY-107414), numidargistat dihydrochloride (HY-101979A), celecoxib (HY-14398), TWS119 (HY-10590), hsa-miR223 mimic (HY-R00467), and miRNA mimic negative control (HY-R04602) were from MedChemExpress (Monmouth Junction, NJ, USA). GSK2795039 (33777) and SCH772984 (19166) were from Cayman Chemical (Ann Arbor, MI, USA). ML385 (S8790), 666-15 (S8846), T5224 (S8966), and PKM2-IN-1 (S8616) were from Selleckchem (Yokohama, Japan). Lipofectamine^®^ RNAiMAX (13778075), SYBR Green qPCR Master Mix (A66732), PGE2 ELISA kit (EHPGE2), SuperSignal West Pico PLUS Chemiluminescent Substrate (34580), and pre-designed/validated siRNAs targeting the negative control (No. 1), ATF2 (ID#: s3492), CREB2/ATF4 (ID#: s1702), CEBPB (ID#: s2893), and K_Ca_3.1 (ID#: s7801) were from Thermo Fisher Scientific (Waltham, MA, USA). IL-4 and IL-13 (human, recombinant, animal free) (AF-200-04, AF-200-13) were from PeproTech Inc (Cranbury, NJ, USA). ReverTra Ace (TRT-101) was from ToYoBo (Osaka, Japan). Flat-bottomed dishes were from Corning (Corning, NY, USA). Random hexanucleotide primer (C1181) was from Promega K.K. (Tokyo, Japan). FastGene RNA Premium kit and FastGene miRNA Enhancer kit were from Nippongenetics (Tokyo, Japan). Mir-X miRNA First-Strand Synthesis kit was from TaKaRa (Osaka, Japan). Arginase Activity Assay kit was from Cosmo Bio (Tokyo, Japan). PCR primers were from Nihon Gene Research Laboratories (Sendai, Japan) (Table S1). HS-F5 column (150 × 2.1 mm; Supelco) was from Merck (Darmstadt, Germany). Primary and secondary antibodies were listed in Table S2. Other chemicals and reagents were from Sigma-Aldrich and FUJIFILM Wako Pure Chemicals.

### 4.2. Cell Culture and Differentiation into M_2_-MACs

The human acute monocytic leukemia cell line THP-1 (RIKEN Cell Bank, Osaka, Japan) was used as a model for human monocyte-derived macrophages. Cells were maintained in RPMI 1640 medium supplemented with 10% FBS and antibiotics at 37 °C in a humidified atmosphere containing 5% CO_2_. To induce macrophage differentiation, THP-1 cells were treated with PMA (100 ng/mL) for 8–12 h. The PMA-containing medium was then removed, and adherent cells were washed and cultured for 72 h in fresh medium supplemented with recombinant human IL-4 and IL-13 (20 ng/mL each) to induce M_2_ polarization. This protocol was based on our previously established M_2_-MAC model [10,11]. Unless otherwise indicated, compounds were dissolved in dimethyl sulfoxide (DMSO), and the final DMSO concentration was matched across experimental groups. M_2_-MACs were exposed to elevated [K^+^]_e_ by adding 30 mM KCl to the culture medium, yielding a final [K^+^]_e_ of approximately 35 mM. Control cells were maintained at the basal [K^+^]_e_ of approximately 5 mM.

### 4.3. RNA Extraction, cDNA Synthesis, and Real-Time PCR

Total RNA was isolated from M_2_-MACs using the acid guanidinium thiocyanate-phenol-chloroform method. RNA concentration and purity were measured using a NanoDrop One microvolume spectrophotometer (Thermo Fisher Scientific). cDNA was synthesized using ReverTra Ace and random hexamers. miRNAs were extracted using the FastGene RNA Premium kit with FastGene miRNA Enhancer, and cDNAs from miRNAs were synthesized using the Mir-X miRNA First-Strand Synthesis kit. Quantitative real-time PCR was performed using an Applied Biosystems 7500 Fast Real-Time PCR System (Thermo Fisher Scientific) and SYBR Green chemistry. PCR primers of human origin are listed in Table S1. ACTB was used as the reference gene, and relative mRNA expression was calculated using the 2^−ΔΔCt^ method. Primer specificity was confirmed by melting-curve analysis.

### 4.4. Western Blotting

Whole-cell lysates were prepared from M_2_-MACs in RIPA buffer supplemented with protease inhibitor cocktails. Protein concentrations were determined using a protein assay kit. Equal amounts of protein were separated by SDS-PAGE and transferred to polyvinylidene difluoride membranes. After blocking, the membranes were incubated sequentially with the primary and secondary antibodies listed in Table S2. Immunoreactive bands were visualized using an enhanced chemiluminescent reagent and imaged with an Amersham Imager 600 (GE Healthcare Japan, Tokyo, Japan). Band intensities were quantified using ImageJ software (Ver. 1.42, National Institutes of Health, NIH, USA) and normalized to the corresponding ACTB signal.

### 4.5. Small Interfering RNA and MicroRNA Transfection

To examine the contribution of transcriptional regulators, M_2_-MACs were transfected with siRNAs targeting CREB2/ATF4, ATF2, CEBPB, or K_Ca_3.1, or with a miR223 mimic. A non-targeting siRNA (control siRNA) and miRNA mimic negative control (control mimic) were used as negative controls. Transfection was performed in adherent cells using Lipofectamine RNAiMAX according to the manufacturer’s instructions. Knockdown efficiency was assessed by real-time PCR.

### 4.6. Measurement of PGE_2_ by Enzyme-Linked Immunosorbent Assay

PGE_2_ concentrations in culture supernatants were measured using a commercially available ELISA kit according to the manufacturer’s instructions. M_2_-MACs were treated with vehicle, SKA-121, elevated [K^+^]_e_, or elevated [K^+^]_e_ plus SKA-121 for 48 h. Culture supernatants were collected, centrifuged to remove cellular debris, and stored at −80 °C until analysis. Absorbance was measured using a SpectraMax384 microplate reader (Molecular Devices Japan, Tokyo, Japan) at 405 nm with a reference wavelength of 580 nm. PGE_2_ concentrations were calculated from a standard curve and expressed relative to the vehicle control. The absolute PGE_2_ concentration in the vehicle control was approximately 250 pg/mL.

### 4.7. Measurement of Amino Acid Concentrations

Culture supernatants were collected after the indicated treatments, centrifuged to remove cell debris, and stored at −80 °C until analysis. L-ARG, L-ORN, and L-CIT were quantified using a Nexera ultra-high-performance liquid chromatography system coupled to an LCMS-8060 triple-quadrupole mass spectrometer equipped with an electrospray ionization source (Shimadzu, Kyoto, Japan) [42]. Chromatographic separation was performed at 40 °C using a Discovery HS-F5 column. Analytes were detected in positive-ion multiple-reaction-monitoring mode using the following transitions: *m/z* 175 > 70 for ARG, *m/z* 133 > 70 for ORN, *m/z* 176 > 70 for CIT, *m/z* 185 > 75 for ARG-^13^C_6_,^15^N_4_, *m/z* 139 > 76 for ORN-d_6_, and *m/z* 180 > 74 for CIT-d_4_. Concentrations were calculated from external standard curves and expressed relative to the vehicle control. Basal concentrations in control M_2_-MAC cultures were approximately 1100 μM ARG, 200 μM ORN, and 20 μM CIT.

### 4.8. Measurement of Arginase Activity

Arginase activity was measured using an Arginase Activity Assay kit according to the manufacturer’s instructions. Briefly, M_2_-MACs were collected after the indicated treatments and lysed. Arginase activity was determined by measuring the amount of urea generated from L-ARG. The enzymatic reaction was initiated by incubating cell lysates with the supplied substrate solution and for the duration specified by the manufacturer. After termination of the reaction, the amount of urea produced was determined colorimetrically using the detection reagents included in the kit. Absorbance (at 540 nm) was measured using a microplate reader, and arginase activity was calculated from a urea standard curve.

### 4.9. Statistical Analyses

Statistical analyses were performed using XLSTAT software (version 2013.1). Differences between two groups were evaluated using paired or unpaired Student’s *t*-tests with Welch’s correction when appropriate. Comparisons among multiple groups were performed using Tukey’s test. A *p* value less than 0.05 was considered statistically significant. Data were presented as the mean ± SEM.

## 5. Conclusions

K_Ca_3.1 activation coordinately suppressed the COX2–PGE_2_ axis and ARG1-dependent ARG catabolism in M_2_-MACs through the ERK–CREB2 transcriptional axis. K_Ca_3.1 activation also attenuated the immunometabolic response to elevated [K^+^]_e_. Thus, K_Ca_3.1 represents a potential immunometabolic checkpoint in macrophages and may provide a pharmacological strategy for reprogramming the immunosuppressive tumor microenvironment. Further studies using in vivo tumor models are required to establish the therapeutic relevance of this pathway.

## Figures and Tables

**Figure 1:**
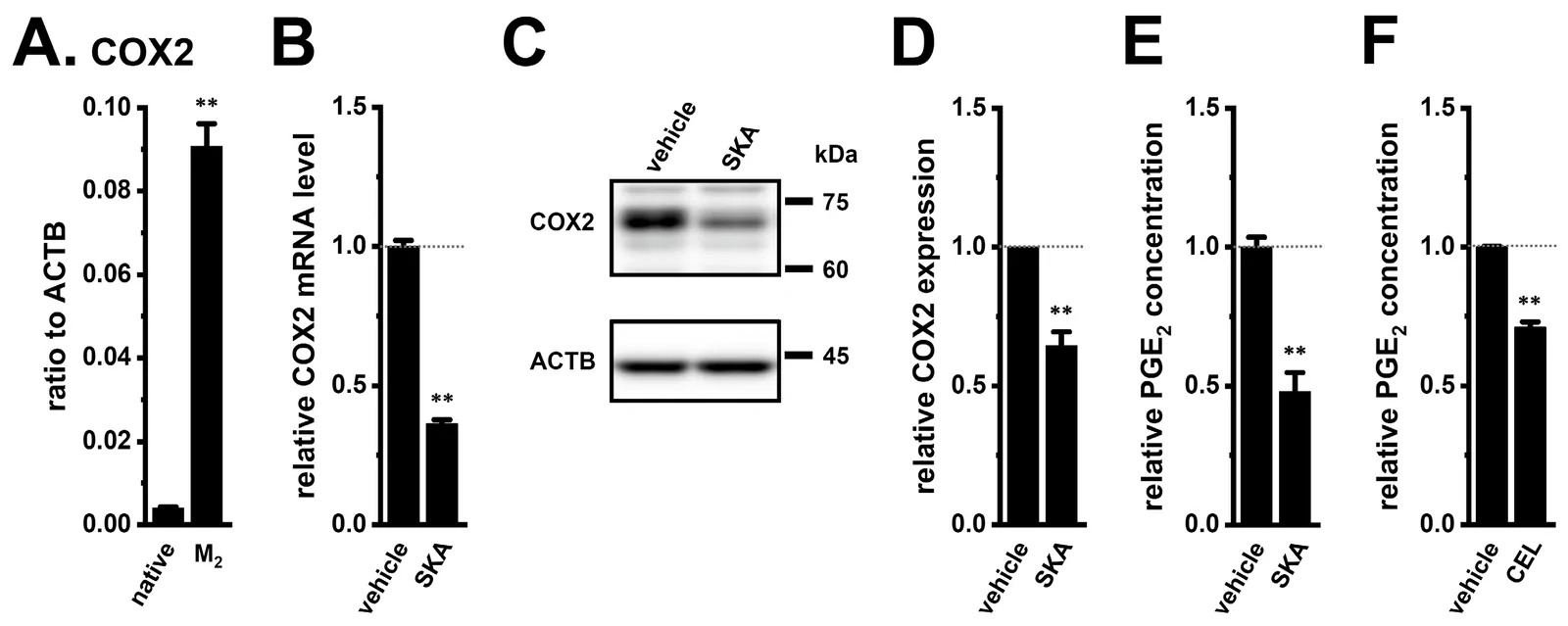
K_Ca_3.1 activation suppresses COX2 expression and PGE_2_ production in M_2_-MACs. (**A**) Real-time PCR examination of the COX2 mRNA expression in undifferentiated THP-1 cells (native) and M_2_-MACs (M_2_). Expression was normalized to ACTB (*n* = 4). (**B**) Real-time PCR examination of the COX2 mRNA expression in M_2_-MACs treated with vehicle (0.1% DMSO) or SKA-121 (SKA; 10 μM) for 24 h. Values were normalized to ACTB and expressed relative to the vehicle control (set to 1.0; *n* = 4). (**C**,**D**) Representative immunoblots and densitometric analysis of COX2 protein in M_2_-MACs treated with vehicle or SKA for 48 h. COX2 signals were normalized to ACTB and expressed relative to the vehicle control (set to 1.0; *n* = 4). The positions of molecular weight markers (kDa) are indicated on the right of each blot (**C**). (**E**,**F**) PGE_2_ concentrations in the culture supernatants from M_2_-MACs treated with vehicle, SKA (**E**), or celecoxib (CEL; 1 μM) (**F**) for 48 h. Values were expressed relative to the vehicle control (set to 1.0; *n* = 4); the absolute concentration in the vehicle control was approximately 250 pg/mL. Data are presented as the mean ± SEM. **: *p* < 0.01 vs. ‘native’ and vehicle control.

**Figure 2:**
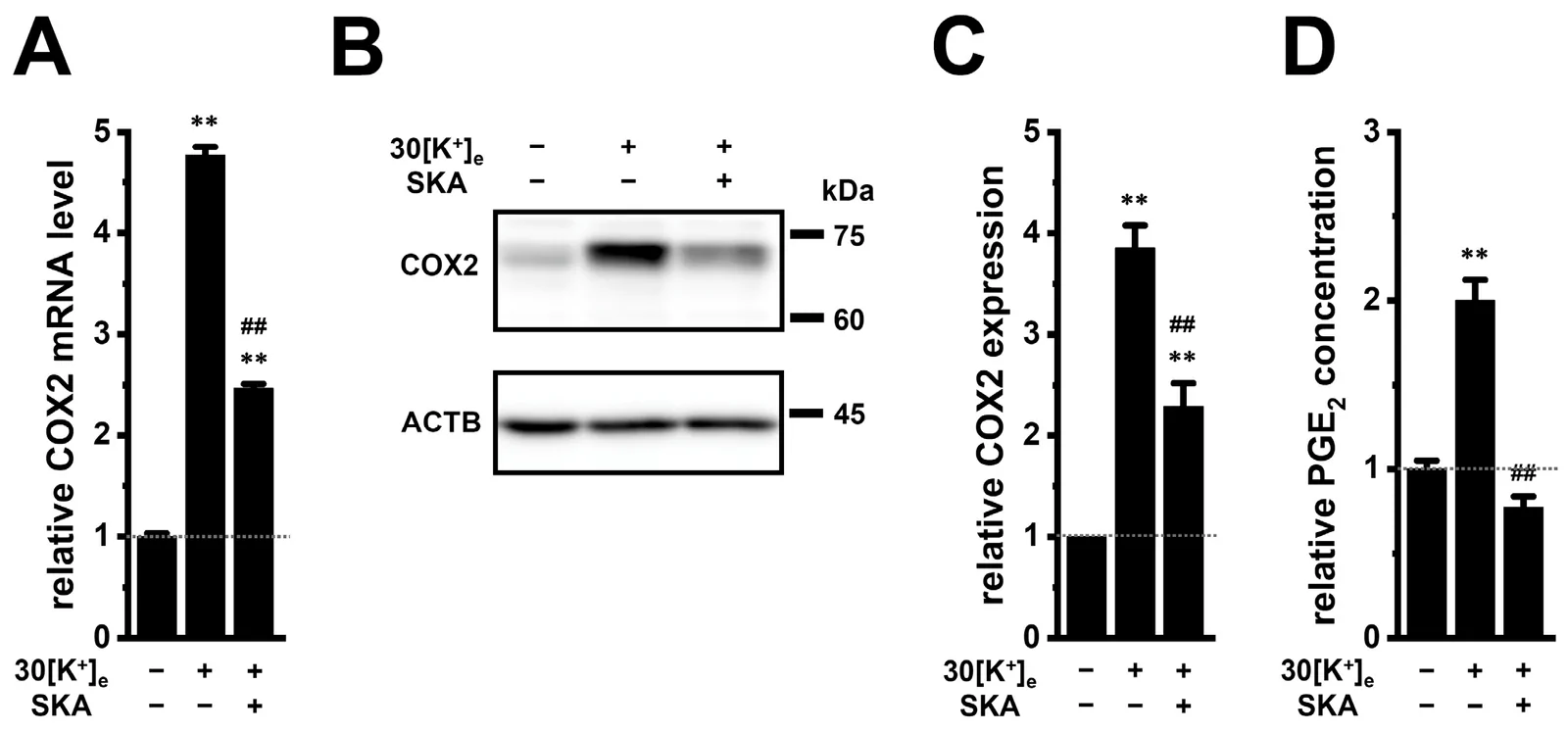
K_Ca_3.1 activation attenuates elevated [K^+^]_e_-induced COX2 expression and PGE_2_ production. COX2 mRNA expression (**A**), representative immunoblots and densitometric analysis of COX2 protein (**B**,**C**), and PGE_2_ production (**D**) in M_2_-MACs cultured under basal [K^+^]_e_ with vehicle (−/−), elevated [K^+^]_e_ induced by addition of 30 mM KCl (+/−; final [K^+^]_e_, approximately 35 mM), or elevated [K^+^]_e_ plus SKA-121 (SKA; 10 μM) (+/+) (*n* = 4). Values were normalized to ACTB and expressed relative to −/− (set to 1.0). The positions of molecular weight markers (kDa) are indicated on the right of each blot (**B**). PGE_2_ concentration was similarly expressed relative to −/−. Data are presented as the mean ± SEM. ^**^: *p* < 0.01 vs. −/−; ^##^: *p* < 0.01 vs. +/−.

**Figure 3:**
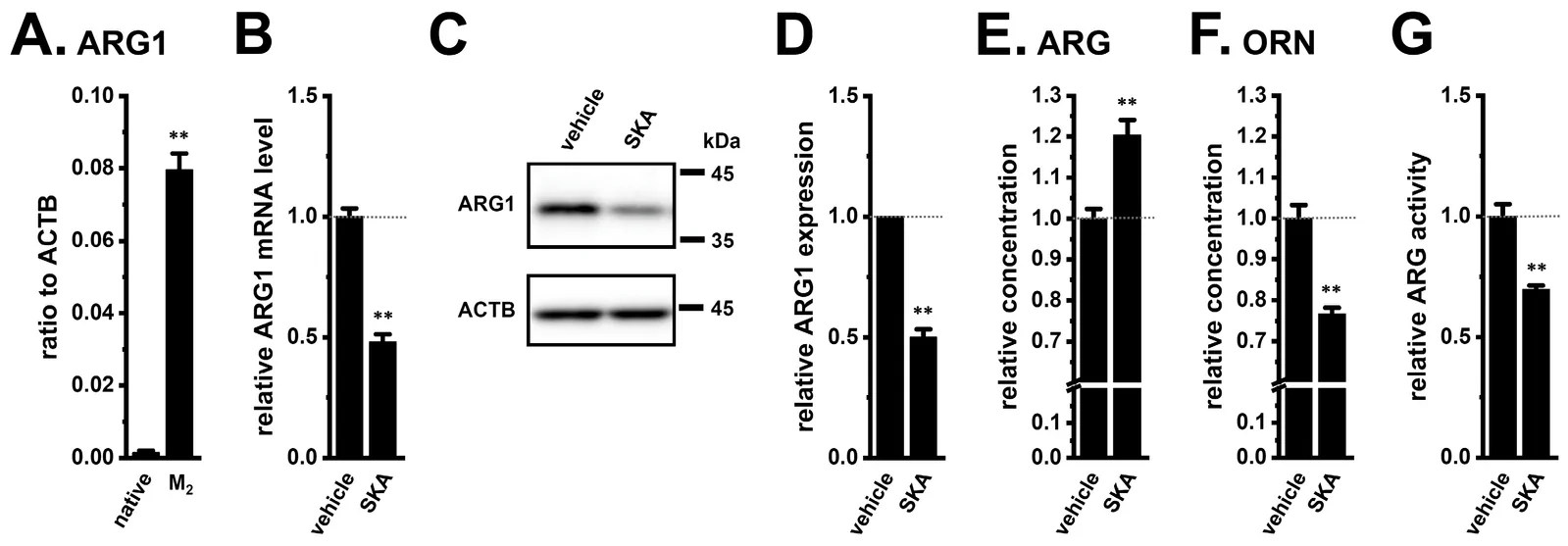
K_Ca_3.1 activation suppresses ARG1 expression and modulates ARG metabolism in M_2_-MACs. (**A**) ARG1 mRNA expression in undifferentiated THP-1 cells (native) and M_2_-MACs (M_2_), measured by real-time PCR and normalized to ACTB (*n* = 4). (**B**) ARG1 mRNA expression in M_2_-MACs treated with vehicle or SKA-121 (SKA; 10 μM) for 24 h. Values were normalized to ACTB and expressed relative to the vehicle control (set to 1.0; *n* = 4). (**C**,**D**) Representative immunoblots and densitometric analysis of ARG1 protein in M_2_-MACs treated with vehicle or SKA for 48 h. The positions of molecular weight markers (kDa) are indicated on the right of each blot (**C**). (**E**,**F**) Relative ARG (**E**) and ORN (**F**) concentrations in the culture supernatants after vehicle or SKA treatment for 48 h. ARG and ORN concentrations were approximately 1200 and 200 μM, respectively. (**G**) Relative arginase activities after vehicle or SKA treatment for 48 h. Relative arginase activities were expressed relative to the vehicle control (set to 1.0; *n* = 4). Data are presented as the mean ± SEM. **: *p* < 0.01 vs. ‘native’ and vehicle control.

**Figure 4:**
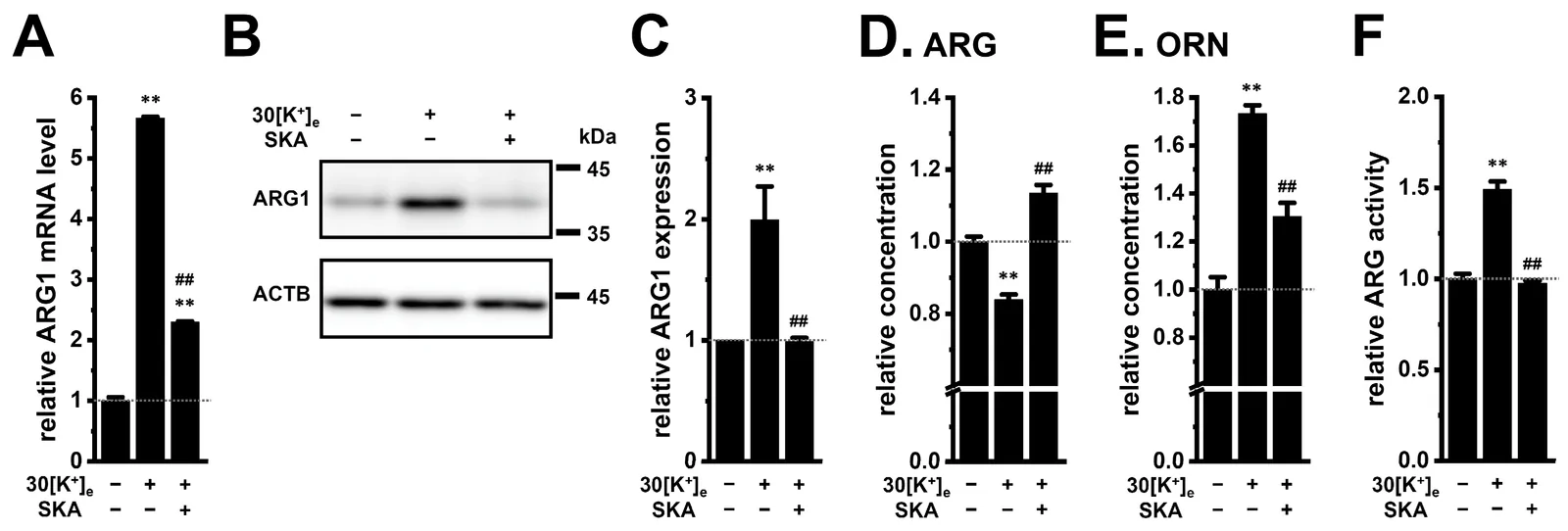
K_Ca_3.1 activation suppresses elevated [K^+^]_e_-induced ARG1 expression and restores ARG/ORN balance. (**A**–**E**) ARG1 mRNA expression (**A**), representative immunoblots and densitometric analysis of ARG1 protein (**B**,**C**), and relative ARG and ORN concentrations (**D**,**E**) in M_2_-MACs cultured under basal [K^+^]_e_ with vehicle (−/−), elevated [K^+^]_e_ (+/−), or elevated [K^+^]_e_ plus SKA-121 (SKA; 10 μM) (+/+) (*n* = 4). The positions of molecular weight markers (kDa) are indicated on the right of each blot (**B**). (**F**) Relative arginase activities after vehicle or SKA treatment for 48 h. Values were normalized to ACTB and expressed relative to vehicle control (set to 1.0). Data are presented as the mean ± SEM. ^**^: *p* < 0.01 vs. −/−; ^##^: *p* < 0.01 vs. +/−.

**Figure 5:**
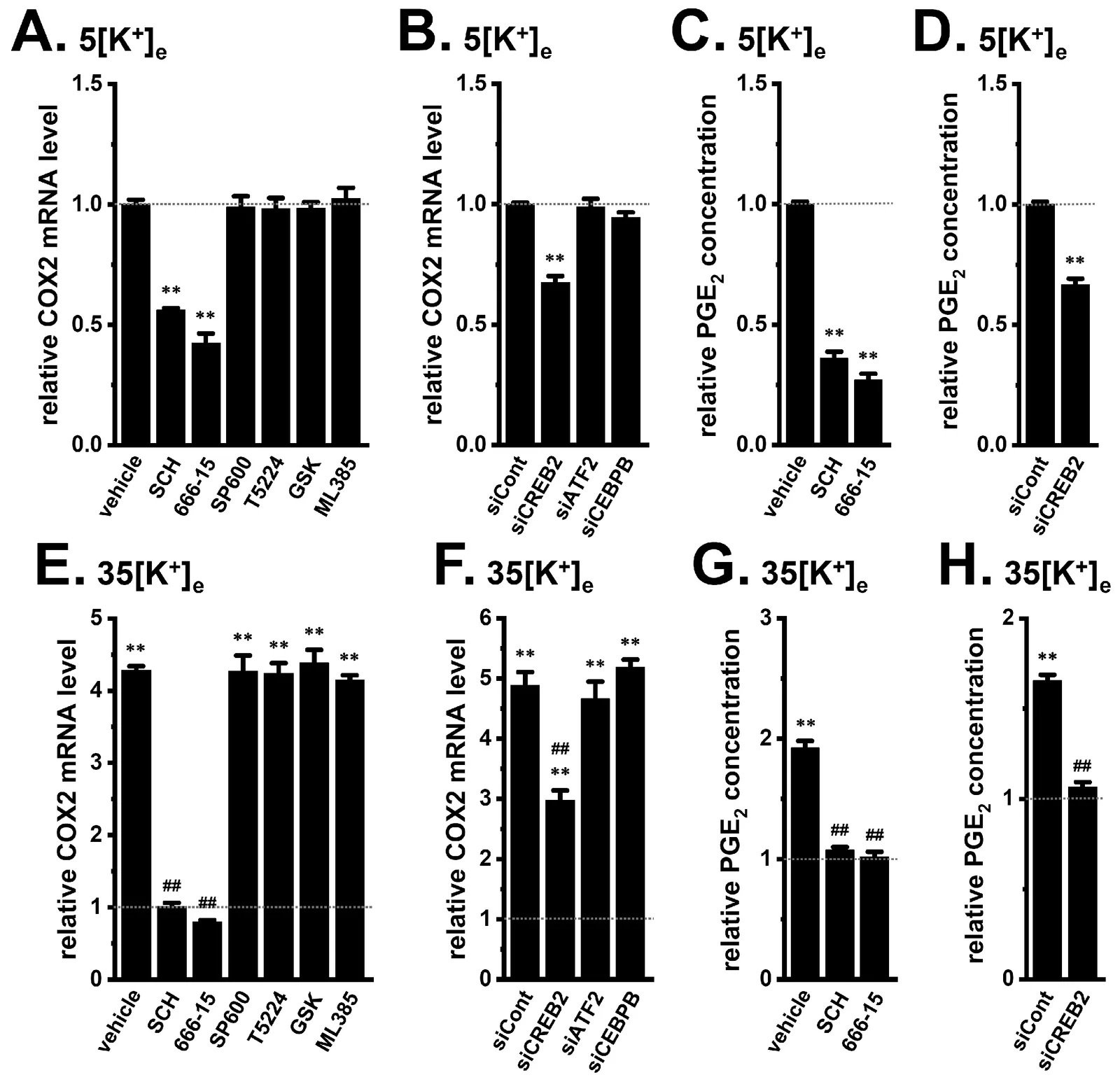
ERK and CREB2 signaling regulate COX2 expression and PGE_2_ production under basal and elevated [K^+^]_e_ conditions. (**A**,**E**) COX2 mRNA expression in M_2_-MACs treated with vehicle, SCH772984 (SCH; 1 μM), 666-15 (1 μM), SP600125 (SP600; 1 μM), T5224 (10 μM), GSK2795039 (GSK; 10 μM), and 5 μM ML385 for 24 h under basal (5 mM) (**A**) and elevated (35 mM) [K^+^]_e_ (**E**) conditions. (**B**,**F**) COX2 mRNA expression in M_2_-MACs transfected with control siRNA (siCont), CREB2 siRNA (siCREB2), ATF2 siRNA (siATF2), and CEBPB siRNA (siCEBPB) under basal (**B**) and elevated [K^+^]_e_ (**F**) conditions. COX2 mRNA expression under basal [K^+^]_e_ conditions was expressed as 1.0 (*n* = 4). (**C**,**G**) PGE_2_ concentrations in M_2_-MACs treated with vehicle, SCH, and 666-15 for 48 h under basal (**C**) and elevated [K^+^]_e_ (**G**) conditions. (**D**,**H**) PGE_2_ concentrations in M_2_-MACs transfected with siCont and siCREB2 under basal (**D**) and elevated [K^+^]_e_ (**H**) conditions. PGE_2_ concentrations under basal [K^+^]_e_ conditions were expressed as 1.0 (*n* = 4). Data are presented as the mean ± SEM. ^**^, ^##^: *p* < 0.01 vs. vehicle controls under basal and elevated [K^+^]_e_, respectively.

**Figure 6:**
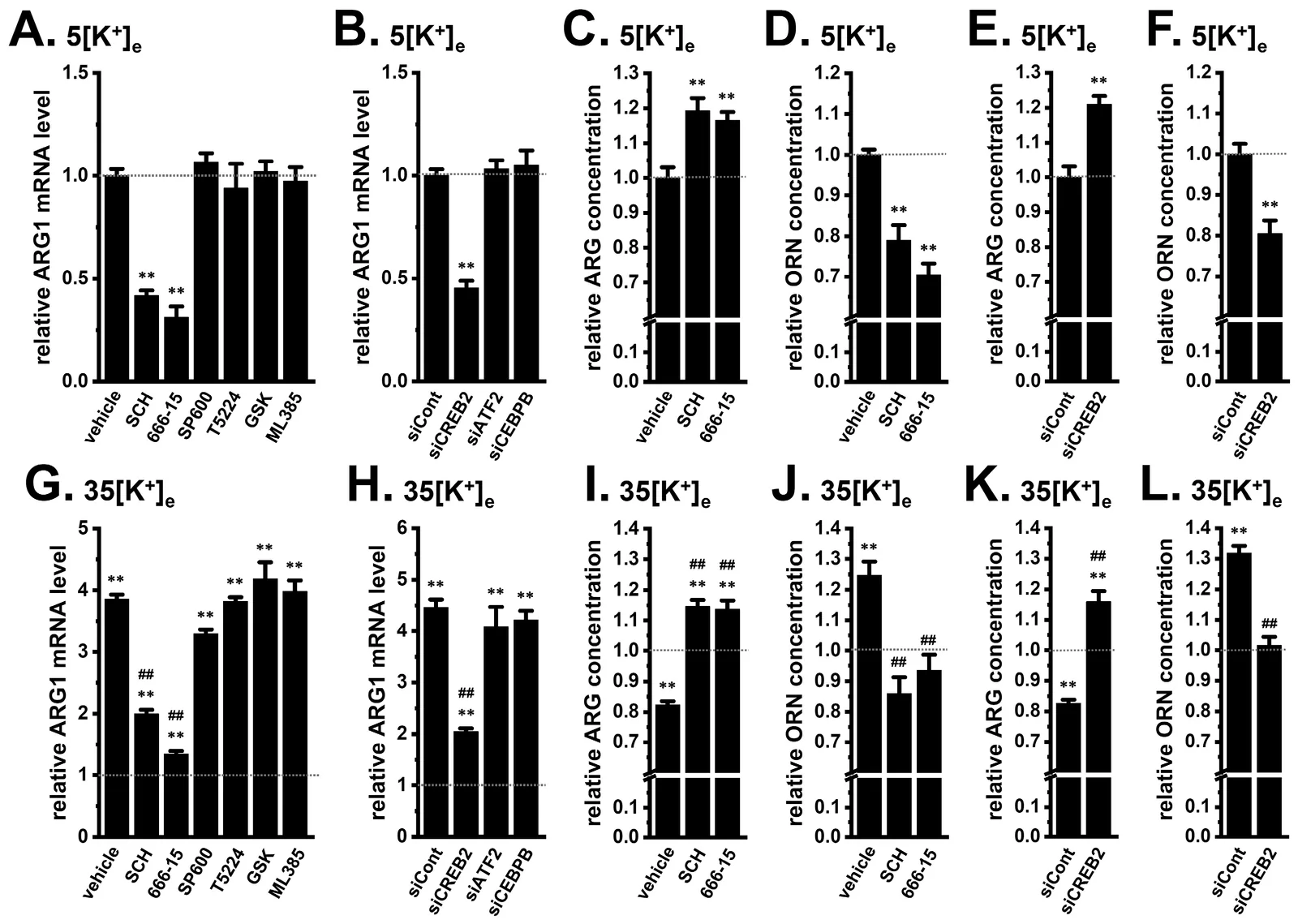
ERK and CREB2 signaling regulate ARG1 expression and restore ARG/ORN balance. (**A**,**G**) ARG1 mRNA expression in M_2_-MACs treated with vehicle, SCH, 666-15, SP600, T5224, GSK, and ML385 for 24 h under basal (**A**) and elevated [K^+^]_e_ (**G**) conditions. (**B**,**H**) ARG1 mRNA expression in M_2_-MACs transfected with siCont, siCREB2, siATF2, and siCEBPB under basal (**B**) and elevated [K^+^]_e_ (**H**) conditions. ARG1 mRNA expression under basal [K^+^]_e_ conditions was expressed as 1.0 (*n* = 4). (**C**,**D**,**I**,**J**) ARG (**C**,**I**) and ORN (**D**,**J**) quantitation in the culture medium of M_2_-MACs treated with vehicle, SCH, and 666-15 for 48 h under basal (**C**,**D**) and elevated [K^+^]_e_ (**I**,**J**) conditions. (**E**,**F**,**K**,**L**) ARG (**E**,**K**) and ORN (**F**,**L**) quantitation in the culture media of M_2_-MACs transfected with siCont and siCREB2 under basal (**E**,**F**) and elevated [K^+^]_e_ (**K**,**L**) conditions. ARG and ORN concentrations under basal [K^+^]_e_ conditions were expressed as 1.0 (*n* = 4). Data are presented as the mean ± SEM. ^**^, ^##^: *p* < 0.01 vs. vehicle control under basal and elevated [K^+^]_e_, respectively.

**Figure 7:**
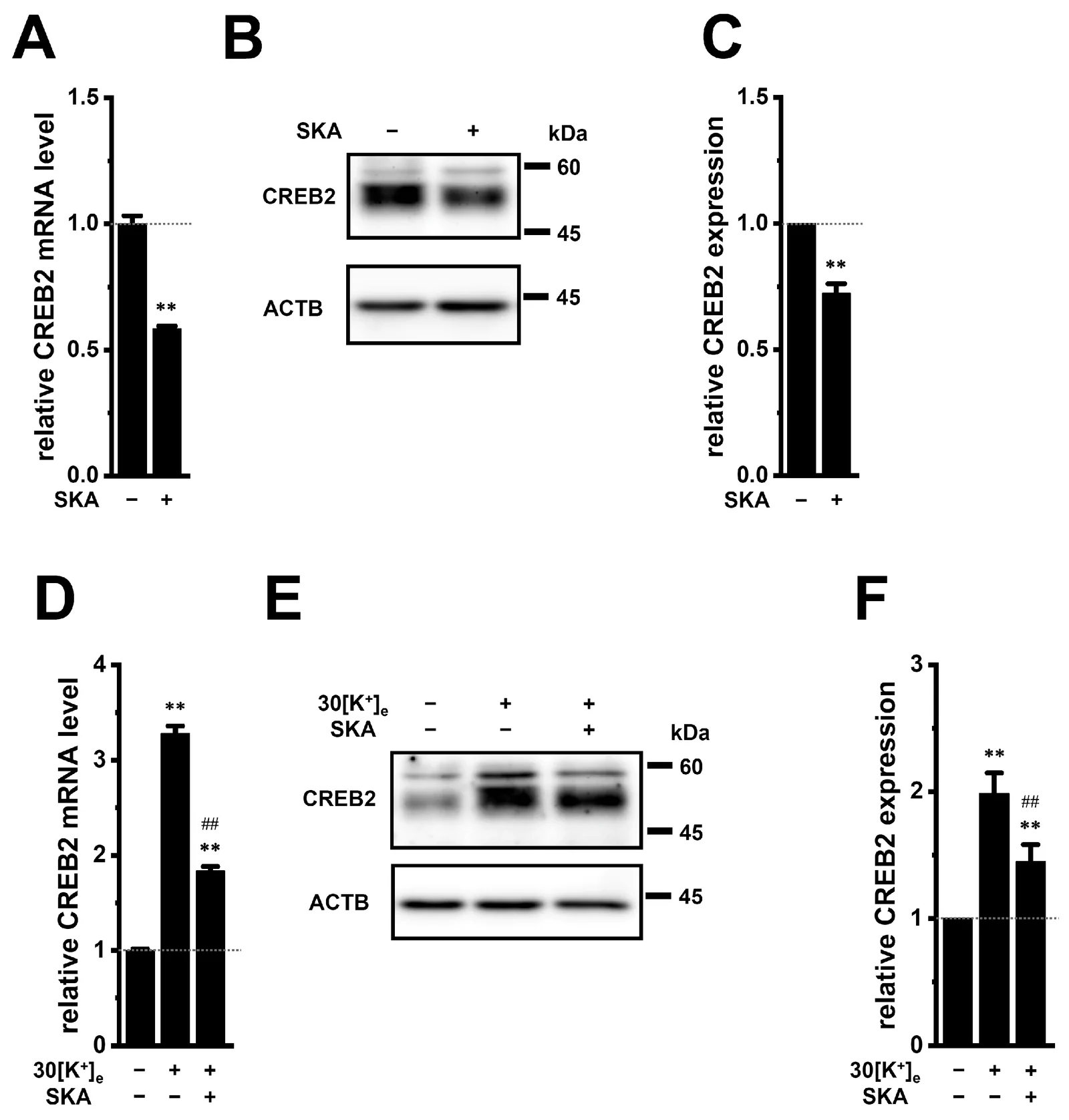
K_Ca_3.1 activation suppresses basal and elevated [K^+^]_e_-induced CREB2 expression in M_2_-MACs. (**A**) Real-time PCR examination of the CREB2 mRNA expression in M_2_-MACs treated with vehicle (−) or SKA-121 (SKA; 10 μM) (+) for 24 h. Values were normalized to ACTB and expressed relative to the vehicle control (set to 1.0; *n* = 4). (**B**,**C**) Representative immunoblots and densitometric analysis of CREB2 protein in M_2_-MACs treated with vehicle (−) or SKA (+) for 48 h. CREB2 signals were normalized to ACTB and expressed relative to the vehicle control (set to 1.0; *n* = 4). CREB2 mRNA expression (**D**) and representative immunoblots and densitometric analysis of CREB2 protein (**E**,**F**) in M_2_-MACs cultured under basal [K^+^]_e_ with vehicle (−/−), elevated [K^+^]_e_ induced by addition of 30 mM KCl (+/−), or elevated [K^+^]_e_ plus SKA-121 (SKA; 10 μM) (+/+) (*n* = 4). The positions of molecular weight markers (kDa) are indicated on the right of each blot (**B**,**E**). Data are presented as the mean ± SEM. ^**^: *p* < 0.01 vs. − and −/−; ^##^: *p* < 0.01 vs. +/−.

## Data Availability

The original data presented in the study are included in the article and Supplementary Materials. Further inquiries may be directed to the corresponding author.

## Supplementary Materials

The following supporting information can be downloaded at: https://www.mdpi.com/article/10.3390/ijms27198477/s1.
