# Supplementary material for: Activation of KCa3.1 K+ Channel Suppresses COX2- and ARG1-Dependent Immunosuppressive Metabolic Programs in THP-1-Derived M2-like Macrophages
Source: Int J Mol Sci. 2026 Sep 23;27(19):8477. doi: 10.3390/ijms27198477 (PMC13665041; doi:10.3390/ijms27198477)
Supplement: Supplementary file 1 [file ijms-27-08477-s001.zip › ijms-4518655-supplementary.pdf]

Supplementary Materials

Activation of $K_{Ca}3.1$ K^+ Channel Suppresses COX2- and ARG1-Dependent Immunosuppressive Metabolic Programs in THP-1-Derived M_2-like Macrophages

Junko Kajikuri[1], Ayano Kitahara[1], Miki Matsui[1], Yasuhiro Maeda[2], Hiroaki Kito[1], Yohei Yamaguchi[1], Ryo Otsuka[1], Susumu Ohya[1,*]

[1] Department of Pharmacology, Graduate School of Medical Sciences, Nagoya City University, Nagoya 467-8601, Japan; kajikuri@med.nagoya-cu.ac.jp (J.K.); kita.aya@med.nagoya-cu.ac.jp (A.K.); c241739@ed.nagoya-cu.ac.jp (M.M.); kito@med.nagoya-cu.ac.jp (H.K.); y_yamagu@med.nagoya-cu.ac.jp (YY); c241905@ed.nagoya-cu.ac.jp (R.O.)
[2] Omics Analysis Laboratory, Open Facility Center, Fujita Health University, Toyoake 470-1192, Japan; ymaeda@fujita-hu.ac.jp

* Correspondence: sohya@med.nagoya-cu.ac.jp; Tel.: +81-52-853-8149

Supplementary Materials: The following are available online at www.mdpi.com/article/doi/s1

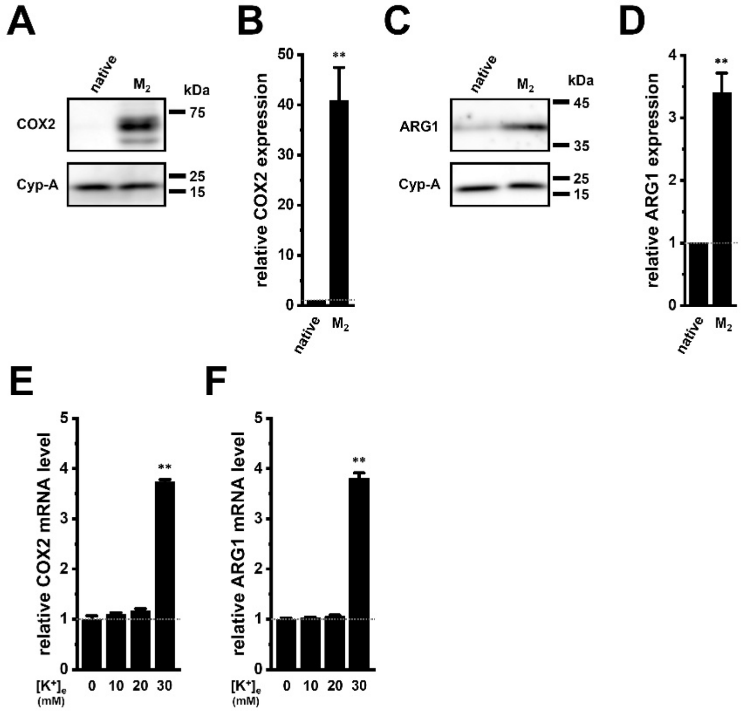

Figure S1. Increased COX2 and ARG1 protein expression during differentiation of THP-1 cells into M_2-MACs and concentration-dependent effects of $[K^+]_e$ elevation on their mRNA expression in M_2-MACs. (**A,C**) Representative immunoblots of COX2 (**A**) and ARG1 (**C**) in undifferentiated THP-1 (native) and M_2-MACs (M_2). Cyclophilin A (Cyp-A) was used as the loading control. (**B,D**) Densitometric analysis of COX2 (**B**) and ARG1 (**D**), normalized to Cyp-A and expressed relative to 'native' (set to 1.0; *n* =4). (**E,F**) Cells were treated with 0, 10, 20, and 30 mM KCl, and the mRNA expression levels were analyzed by real-time PCR. Expression levels were normalized to ACTB and are shown relative to 0 mM $[K^+]_e$ (set to 1.0). Data are presented as the means ± SEM. **: $p < 0.01$ vs. 'native' and 0 mM $[K^+]_e$.

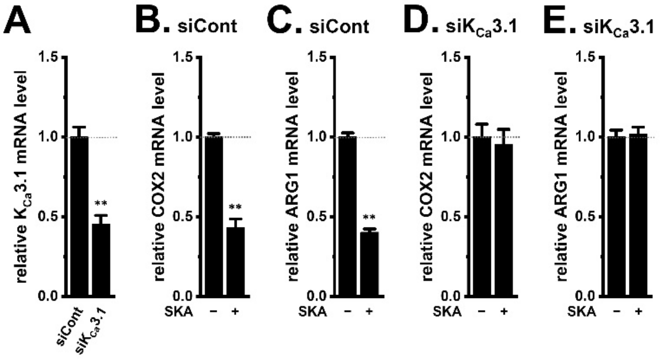

Figure S2. Effects of siRNA-mediated inhibition of $K_{Ca}3.1$ on SKA-121-induced downregulation of COX2 and ARG1 in M_2-MACs. (**A**) Real-time PCR analysis of $K_{Ca}3.1$ expression in M_2-MACs transfected with the respective control siRNA (siCont) or $K_{Ca}3.1$ siRNA ($siK_{Ca}3.1$) for 48 h (*n* = 4). (**B-E**) Real-time PCR analysis of COX2 (**B,D**) and ARG1 (**C,E**) expression in M_2-MACs transfected with siCont or $siK_{Ca}3.1$ in the absence (–) and presence (+) of 10 μM SKA-121 (SKA) for 48 h (*n* = 4). Expression levels were normalized to ACTB and are shown relative to siCont and vehicle control (–) (set to 1.0). Data are presented as the means ± SEM. **: $p < 0.01$ vs. siCont and –.

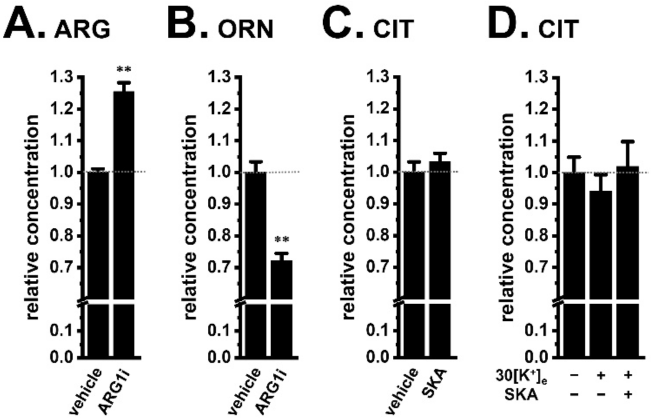

Figure S3. Effects of ARG1 inhibition, $K_{Ca}3.1$ activation, and elevated $[K^+]_e$ on ARG metabolites in M_2-MACs. (**A**,**B**) Relative ARG (**A**) and ORN (**B**) concentrations in culture supernatants from M_2-MACs treated with vehicle or ARG1 inhibitor numidargistat (ARG1i; 10 μM) for 48 h ($n = 4$). (**C**) Relative CIT concentration after treatment with vehicle or SKA-121 (SKA; 10 μM) for 48 h ($n = 4$). (**D**) Relative CIT concentration in M_2-MACs cultured under basal (–/) or elevated (+/) $[K^+]_e$, in the absence (/–) or presence (/+) of SKA-121 for 48 h ($n = 4$). Values are expressed relative to the respective vehicle controls (–/–) (set to 1.0). Data are presented as the means ± SEM. **: $p < 0.01$ vs. –/–.

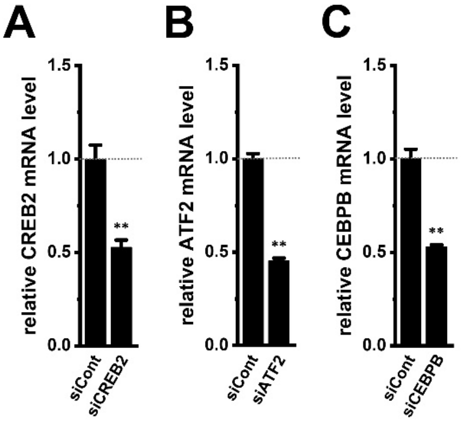

Figure S4. Knockdown efficiencies of CREB2, ATF2, and CEBPB siRNAs in M_2-MACs. Real-time PCR analysis of CREB2 (**A**), ATF2 (**B**), and CEBPB (**C**) expression in M_2-MACs transfected with the respective control siRNA (siCont) or target-specific siRNA (siCREB2, siATF2, or siCEBPB) for 48 h ($n = 4$). Expression levels were normalized to ACTB and are shown relative to siCont-transfected cells (set to 1.0). Data are presented as the means ± SEM. **: $p < 0.01$ vs. siCont.

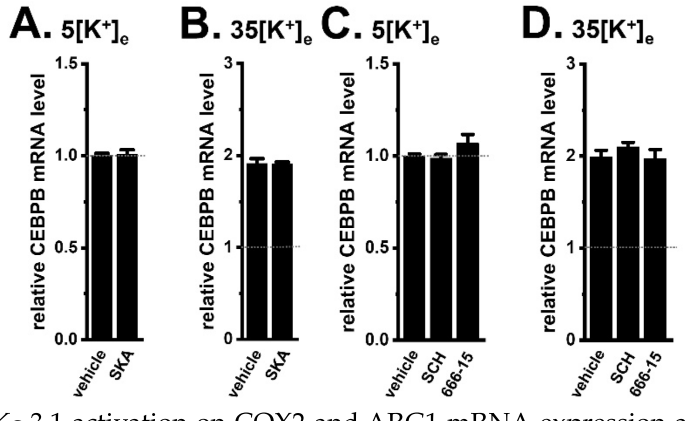

Figure S5. Effects of $K_{Ca}3.1$ activation on COX2 and ARG1 mRNA expression and effects of ERK and CREB inhibition on CEBPB mRNA expression in M_2-MACs under basal and elevated $[K^+]_e$ conditions.

(**A,B**) Real-time PCR analysis of CEBPB expression in M_2-MACs exposed to basal (**A**) or elevated (**B**) $[K^+]_e$, respectively, and treated with vehicle or SKA-121 (SKA; 10 μM) for 24 h (*n* = 4). (**C,D**) CEBPB mRNA expressions in M_2-MACs under basal (**C**) or elevated (**D**) $[K^+]_e$ and treated with vehicle, 1 μM SCH772984 (SCH), or 1 μM 666-15 for 24 h (*n* = 4). Data are presented relative to the vehicle control under basal $[K^+]_e$ (set to 1.0). Data are shown as the means ± SEM.

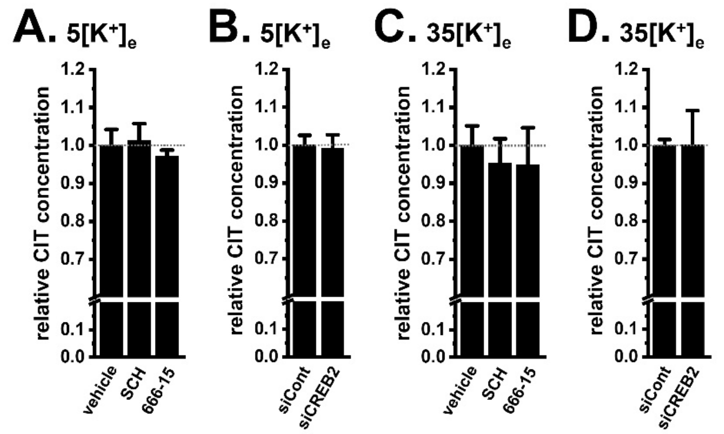

Figure S6. Effects of inhibition of ERK–CREB2 signaling on CIT concentrations in M_2-MACs. (**A,C**) Relative CIT concentrations in the culture supernatants from M_2-MACs under basal (**A**) and elevated (**C**) $[K^+]_e$ conditions and treated with vehicle, 1 μM SCH772984 (SCH), or 1 μM 666-15 for 48 h (*n* = 4). (**B,D**) Relative CIT concentrations in M_2-MACs transfected with siCont or siCREB2 under basal (**B**) and elevated (**D**) $[K^+]_e$ conditions (*n* = 4). Concentrations are shown relative to the corresponding vehicle- or siCont-treated group (set to 1.0). Data are presented as the means ± SEM.

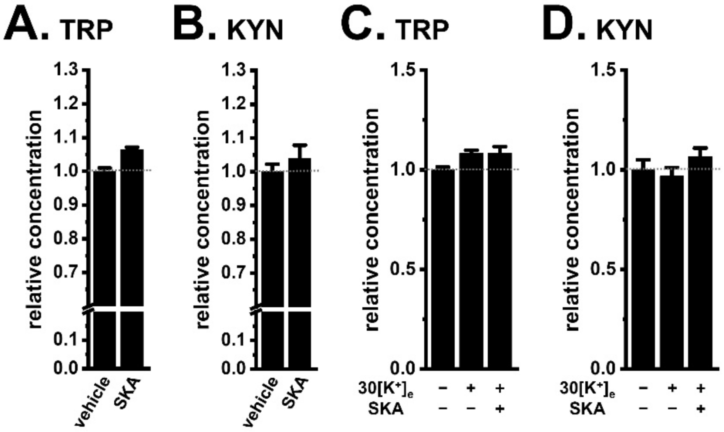

Figure S7. Effects of $K_{Ca}3.1$ activation on tryptophan and kynurenine concentrations in M_2-MACs under basal and elevated $[K^+]_e$ conditions. (**A,B**) Relative concentrations of tryptophan (TRP) (**A**) and kynurenine (KYN) (**B**) in the culture supernatants from M_2-MACs treated with vehicle or SKA-121 (SKA; 10 μM) for 48 h (*n* = 4) under basal $[K^+]_e$ conditions. (**C,D**) Relative TRP (**C**) and KYN (**D**) concentrations in M_2-MACs under elevated $[K^+]_e$ conditions, in the absence (+/–) or presence (+/+) of SKA for 48 h (*n* = 4). Concentrations are shown relative to the vehicle control under basal $[K^+]_e$ (–/–) (set to 1.0). Data are presented as the means ± SEM.

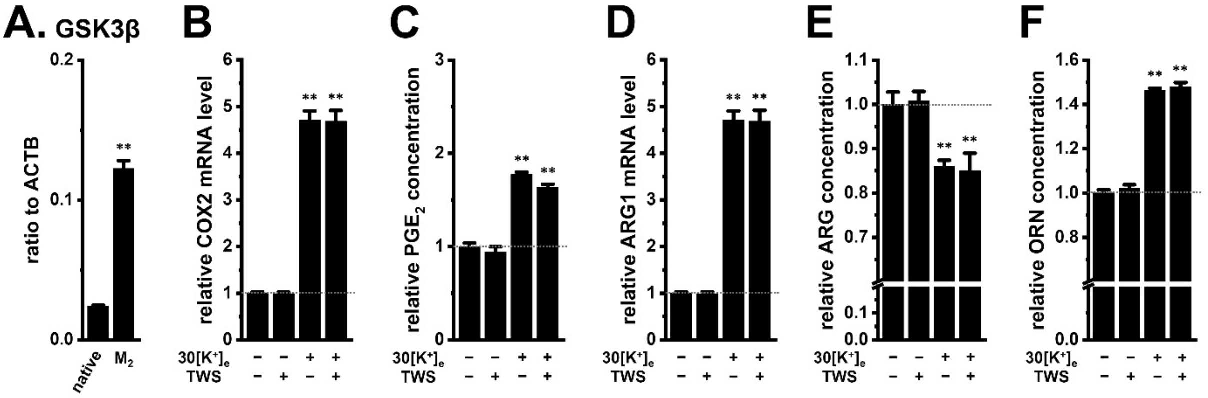

Figure S8. GSK3β inhibition does not affect elevated [K+]e-induced COX2 and ARG1 expression in M2-MACs. (**A**) Real-time PCR analysis of the GSK3β mRNA expression in undifferentiated THP-1 cells (native) and M2-MACs (M2). GSK3β expression was normalized to ACTB (n =4). (**B,D**) Real-time PCR analysis of COX2 (**B**) and ARG1 (**D**) in M2-MACs treated with vehicle or TWS119 (TWS; 1 μM) for 24 h under basal or elevated $[K^+]_e$ conditions (n = 4). Data are shown relative to the vehicle control under basal $[K^+]_e$ (–/–) (set to 1.0). (**C,E,F**) Relative PGE_2 (**C**), ARG (**E**), and ORN (**F**) concentrations in the culture supernatants from M2-MACs under basal and elevated $[K^+]_e$ conditions and treated with vehicle or TWS for 48 h (n = 4). Concentrations are shown relative to the corresponding vehicle control under basal $[K^+]_e$ (–/–) (set to 1.0). Data are presented as the means ± SEM. **: $p < 0.01$ vs. –/–.

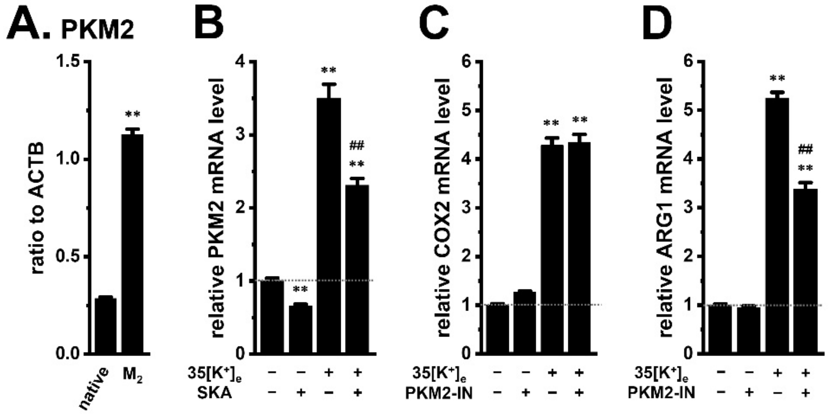

Figure S9. $K_{Ca}3.1$ activation upregulates PKM2 expression but PKM2 inhibition does not suppress COX2 expression under basal and elevated $[K^+]_e$ conditions in M2-MACs. (**A**) Real-time PCR analysis of the PKM2 mRNA expression in undifferentiated THP-1 cells (native) and M2-MACs (M2) (n =4). (**B**) PKM2 mRNA expression in M2-MACs treated with vehicle and SKA-121 (SKA; 10 μM) for 24 hr under basal and elevated $[K^+]_e$ conditions. (**C,D**) COX2 (**C**) and ARG1 (**D**) mRNA expression in M2-MACs treated with vehicle or PKM2-IN-1 (PKM2-IN; 100 μM) for 24 h under basal or elevated $[K^+]_e$ conditions (n =4). Values were normalized to ACTB and expressed relative to the basal $[K^+]_e$ conditions (–/–) (set to 1.0). Data are presented as the means ± SEM. **,##: $p < 0.01$ vs. –/–, +/–, respectively.

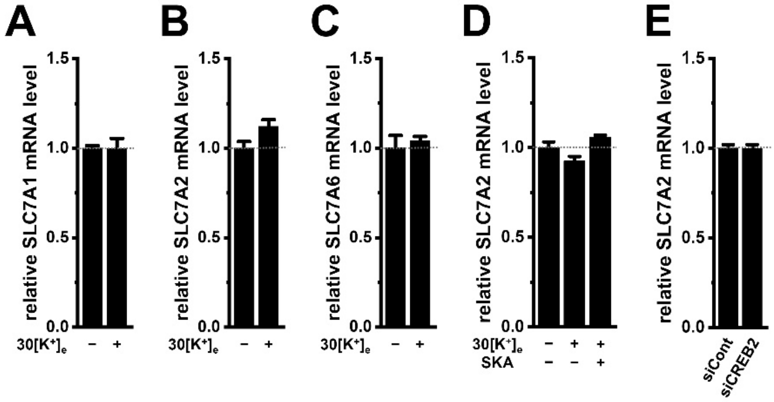

Figure S10. Elevated $[K^+]_e$ exposure and $K_{Ca}3.1$ activation do not substantially alter the expression of arginine transporter SLC7A isoforms in M_2-MACs. (**A,B,C**) Real-time PCR analysis of the SLC7A1 (**A**), SLC7A2 (**B**), and SLC7A6 (**C**) mRNA expressions in M_2-MACs exposed to basal (–) and elevated (+) $[K^+]_e$ conditions. (**D**) Real-time PCR analysis of the SLC7A2 mRNA expression in M_2-MACs treated with vehicle and SKA-121 (SKA; 10 μM) for 24 h under basal and elevated $[K^+]_e$ conditions. The expression levels under basal $[K^+]_e$ (–) were expressed as 1.0 (n = 4). (**E**) SLC7A2 mRNA expressions in M_2-MACs transfected with control siRNA (siCont) and CREB2 siRNA (siCREB2) under basal $[K^+]_e$ conditions. SLC7A2 mRNA level in siCont group was expressed as 1.0 (n = 4). Data are presented as the means ± SEM.

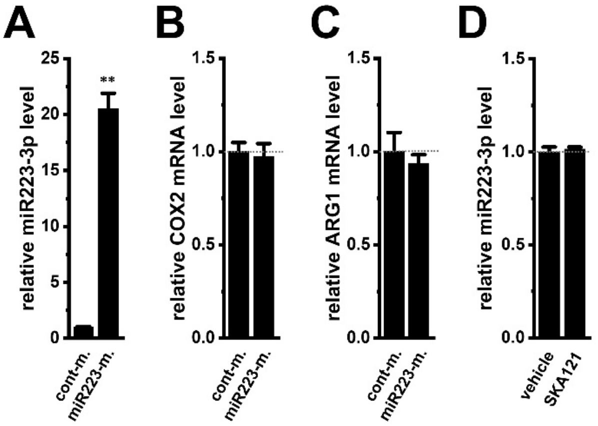

Figure S11. Effects of miR223-3p mimic on the expression levels of COX2 and ARG1 and effects of SKA-121 on the expression levels of miR223-3p in M_2-MACs. (**A**) Real-time PCR of miR223-3p in M_2-MACs transfected with control mimic (control-m.) and miR223-3p mimic (miR223-m.) for 48 h. Expression was normalized to U6 (n = 4). (**B,C**) Real-time PCR of COX2 (**B**) and ARG1 (**C**) in them (n = 4). Expression was normalized to ACTB (n = 4). (**D**) Real-time PCR of miR223-3p in M_2-MACs treated with vehicle and SKA121 (SKA; 10 μM) for 24 hr. Expression was normalized to U6 (n = 4). **: $p < 0.01$ vs. cont-m.

Table S1. List of PCR primers used in this study

Target name	Genbank accession number	Primer sequence		Amplicon length (bp)
		Forward (5' to 3')	Reverse (5' to 3')	
COX2	NM_000963	ACAGCGTAAACTGCGCCTTT	GGGACTTGAGGAGGGTAGATCA	120
ARG1	NM_001244438	ACAAAACAGGGCTACTCTCAGGAT	CCGAGCAAGTCCGAAACAAG	131
$K_{Ca}3.1$	NM_002250	AGCTCCGGGAACAAGTGAAC	GCCAGCGTGTCAATCTGTTT	121
CREB2/ATF4	NM_001675	GTGGCCAAGCACTTCAAACC	GAAGGCATCCTCCTTGCTGTT	120
ATF2	NM_001880	CAAACCATGCCTGTTGCTATTC	AGGACCTGGGATTCCTGGAA	120
CEBPB	NM_001285878	GCCCTCGCAGGTCAAGAG	TGCGCACGGCGATGT	107
GSK3β	EU302497	CGTCAGGAACAGGACATTTCAC	GTGGTGTTAGTCGGGCAGTTG	120
PKM2	NM_002654	AGTACCATGCGGAGACCATCA	CAGTTCGGATCTCAGGTCCTTT	120
SLC7A1	NM_003045	ATTCATGCCCTTCGGGTTCT	ATGGCCTTCTGTGGGTTCTTC	120
SLC7A2	NM_003046	ACCGGTTTGCGACAGCAA	CTGTCCAGGGTCACGATTTTTC	120
SLC7A3	NM_032803	GAGACTCGCCTTGCCAGATG	TGGCCCTGCTTTATCTTTGG	120
SLC7A6	NM_001076785	TGCATATGCTCCGTGTTTCTG	TGGCAGGTAAACACCCATGA	120
ACTB	NM_001101	AGGCCAACCGCGAGAAGATG	GCCAGAGGCGTACAGGGATA	101
has-miR223-3p		TGTCAGTTTGTCAAATACC		
U6		CTCGCTTCGGCAGCACA	AACGCTTCACGAATTTGCGT	

Table S2. List of antibodies used in this study

Type	Antibody name	Host species	Working dilution	Company	Product code	Observed MW (approx. kDa)
Primary antibody	cyclooxygenase-2 (COX2)	rabbit polyclonal	1:3000	ProteinTech (Rosemont, MO, USA)	12375-1-AP	70
	arginase-1 (ARG1)	rabbit polyclonal	1:6000	ProteinTech	16001-1-AP	40
	CREB2 / ATF4	rabbit polyclonal	1:1000	ProteinTech	10835-1-AP	50
	β-actin (ACTB)	mouse monoclonal	1:15000	Sigma-Aldrich (St Louis, MO, USA)	A1978	45
	cyclophilin A (Cyp-A)	mouse monoclonal	1:8000	ProteinTech	67880-1-Ig	20
Secondary antibody	HRP-conjugated anti-mouse IgG	goat polyclonal	1:15000	ThermoFisher Scientific (Waltham, MA, USA)	31430	
	HRP-conjugated anti-rabbit IgG	goat polyclonal	1:7500	ThermoFisher Scientific	AP307P	

MW: molecular weight, HRP: horseradish peroxidase.
